# Caesarean Scar Pregnancy Presenting With Septic Shock and Multiorgan Dysfunction in a High-Risk Cardiac Patient: A Case Report

**DOI:** 10.7759/cureus.86293

**Published:** 2025-06-18

**Authors:** Soumya S Patil, Vidya M Jadhav

**Affiliations:** 1 Obstetrics and Gynaecology, Bharati Vidyapeeth (Deemed to Be University) Medical College and Hospital, Sangli, IND

**Keywords:** caesarean scar, multiorgan dysfunction, pregnancy, rheumatic heart disease, septic shock

## Abstract

Caesarean scar pregnancy (CSP) is a rare form of ectopic pregnancy characterised by the implantation of the gestational sac at the site of a previous caesarean section scar. With the global increase in caesarean delivery rates, the incidence of CSP has been rising correspondingly. We present a case of a 28-year-old woman with an undiagnosed CSP and approximately eight weeks of amenorrhea, who presented in septic shock, further complicated by preexisting cardiac comorbidities. This case highlights the critical importance of prompt recognition, timely diagnosis, and coordinated multidisciplinary management to optimise outcomes and decrease severe maternal morbidity and mortality, and also strongly discourages self-administration of medical termination of pregnancy (MTP) pills, especially in the absence of clinical evaluation and imaging in pregnant women.

## Introduction

Caesarean scar pregnancy (CSP) is a rare but potentially life-threatening form of ectopic pregnancy where the blastocyst implants into a previous lower uterine segment caesarean section scar. The increasing global rate of caesarean deliveries has led to a concurrent rise in the incidence of CSP [1,2]. Early diagnosis and prompt intervention are crucial to prevent severe maternal morbidity and mortality, which can result from uterine rupture, massive haemorrhage, or infection. CSP poses an even greater risk when complicated by comorbid conditions such as rheumatic heart disease (RHD) and pulmonary arterial hypertension (PAH), which further compromise maternal hemodynamic stability [3]. This case report highlights a complex clinical presentation of CSP in a young woman with underlying cardiac pathology and septic shock, illustrating the diagnostic, therapeutic, and ethical challenges of managing such high-risk pregnancies.

## Case presentation

Case history

A 28-year-old, unbooked G2P1L1 woman, presented to the emergency department with complaints of vaginal bleeding, abdominal and back pain for 24 days, and breathlessness for 14 days. She had a history of self-administering medical termination of pregnancy (MTP) pills 24 days earlier. She took mifepristone 200 mg orally and misoprostol 200 mcg (four tablets). On the same day, after self administration, she developed vaginal bleeding and abdominal pain. We are presenting the case presentation in SBAR (Situation, Background, Assessment, and Recommendation) format for easy reading.

Situation

The patient presented to emergency department with severe vaginal bleeding, worsening abdominal pain, and breathlessness on day 10 after self-administration of the pills, at a government hospital (community health centre). On history taking from the patient, the doctor at the government hospital requested some investigation for the same. However, the patient went against medical advice, and details of the treatment provided there are unavailable.

Background

On the 21st day after taking the pills, she consulted a private hospital for similar complaints, as symptoms were worsening. At that time, a pelvic ultrasound, pelvic MRI, and echocardiography were performed for the above symptoms. The 2D-echocardiography findings showed an ejection fraction (EF) of 60%, right ventricular systolic pressure (RVSP) of 90 mmHg, and severe mitral stenosis (MS). She was diagnosed with PAH, severe MS due to RHD, heart failure with preserved ejection fraction (HFpEF), hyperbilirubinemia, and a chronic scar from ectopic pregnancy. She was symptomatically treated and was then referred to our centre. Her past menstrual cycles had been regular, and she had experienced two months of amenorrhea. Regarding her obstetric history, she had one male child, aged four, delivered by caesarean section due to cephalopelvic disproportion.

Assessment

Upon admission to our centre, the patient’s general condition was poor, though she was conscious and oriented. She was tachycardic, with a heart rate of 140 beats per minute, and tachypenic with 31 breaths per minute and had a blood pressure of 110/70 mmHg, with normal oxygen saturation on room air. Her temperature at admission was 36.9 degrees Celsius. Cardiovascular examination revealed a diastolic murmur, loud P2, and a loud S1. Respiratory examination showed equal bilateral air entry with crepitations. Abdominal examination revealed a soft, non-tender, non-distended abdomen; the abdominal scar of the previous lower segment caesarean section (LSCS) was visible but non-tender. Per speculum examination revealed a brownish, foul-smelling discharge. On per vaginal examination, the cervical os was closed; the uterus was anteverted, anteflexed, bulky, and tender on bimanual palpation.

Recommendations

**Figure 1:**
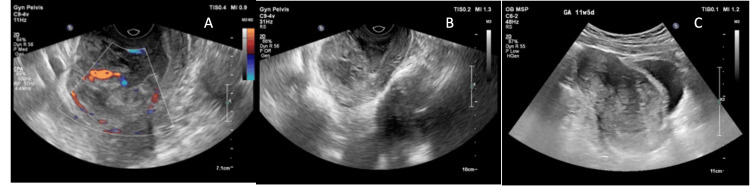
(A) Heterogenous lesion with enhanced vascularity on ultrasonography; (B) empty uterine cavity; (C) thinning of myometrial wall.

Urgent ultrasonography was repeated (Figures 1A-1C), showing a gestational sac implanted within the previous caesarean scar. Transvaginal ultrasonography findings included (i) an empty uterine cavity; (ii) a heterogeneous lesion measuring approximately 64 × 48 × 46 mm involving the anterior uterine wall at the lower uterine segment; (iii) increased vascularity within the lesion (trophoblastic flow); and (iv) thinning of the myometrium, with a normal bladder wall. A hematoma was noted in the cervical canal. The final impression was reported as a scar ectopic pregnancy type 2.

Other Investigations

Beta-human chorionic gonadotropin (β-hCG) values showed a declining trend with an initial level of 8079 mIU/mL done at a private clinic, dropping to 5453 mIU/mL following admission at our centre in a 48-hour period (Table 1).

**Table 1:** Laboratory results. Hb: haemoglobin; PRBC: packed red blood cells; WBC: white blood cells; BUL: blood urea level; SGOT: serum glutamic oxaloacetic transaminase; SGPT: serum glutamic pyruvate transaminase; PT: prothrombin time; INR: international normalised ratio

Parameters	Pre-operative	Post-operative
Hb	11.5 mg/dL (2 PRBC transfused outside hospital at Hb 8.4 mg/dL)	10.9 mg/dL
WBC	13900/mm^3^	67900/mm^3^
Platelets	344000/mm^3^	435000/mm^3^
Blood sugar	80 mg/dL	-
BUL	25 mg/dL	49 mg/dL
Serum creatinine	0.6 mg/dL	2.1 mg/dL
Serum Na+	133 mmol/L	157 mmol/L
Serum K+	4.1 mmol/L	6.1 mmol/L
Serum calcium	-	5.4 mg/dL
SGOT	19 units/L	10458 units/L
SGPT	26 units/L	5982 units/L
Total bilirubin	3 mg/dL	3.4 mg/dL
Direct bilirubin	0.8mg/dL	2.3 mg/dL
Indirect bilirubin	2.20 mg/dL	1.1 mg/dL
Serum proteins	6.9 gm/dL	5.7 gm/dL
Serum albumin	4.1 gm/dL	3.1 gm/dL
Serum globulin	2.8 gm/dL	2.60 gm/dL
PT/INR	11.9 sec/1.00	34.3 sec/2.94

Intervention and management

A high-risk balloon mitral valvotomy (BMV) was planned by the cardiology team upon the clinical stabilisation of the patient. Informed consent was obtained for the excision of the scar ectopic pregnancy and/or emergency hysterectomy if required. Consent for the high-risk intervention, including the risk of intra-procedural death, was also duly obtained. An emergency exploratory laparotomy was performed under general anaesthesia.

Intraoperative Findings

**Figure 2:**
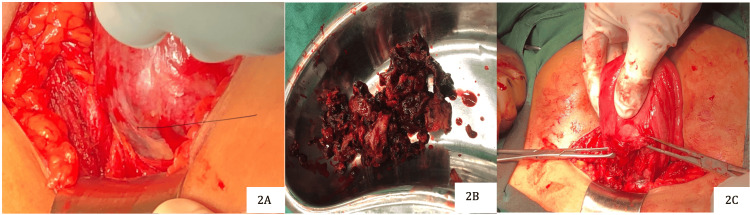
(2A) Bulging of products of conception through the previous uterine scar site; (2B) products of conception with hematoma; (2C) scar site after removing products.

**Figure 3:**
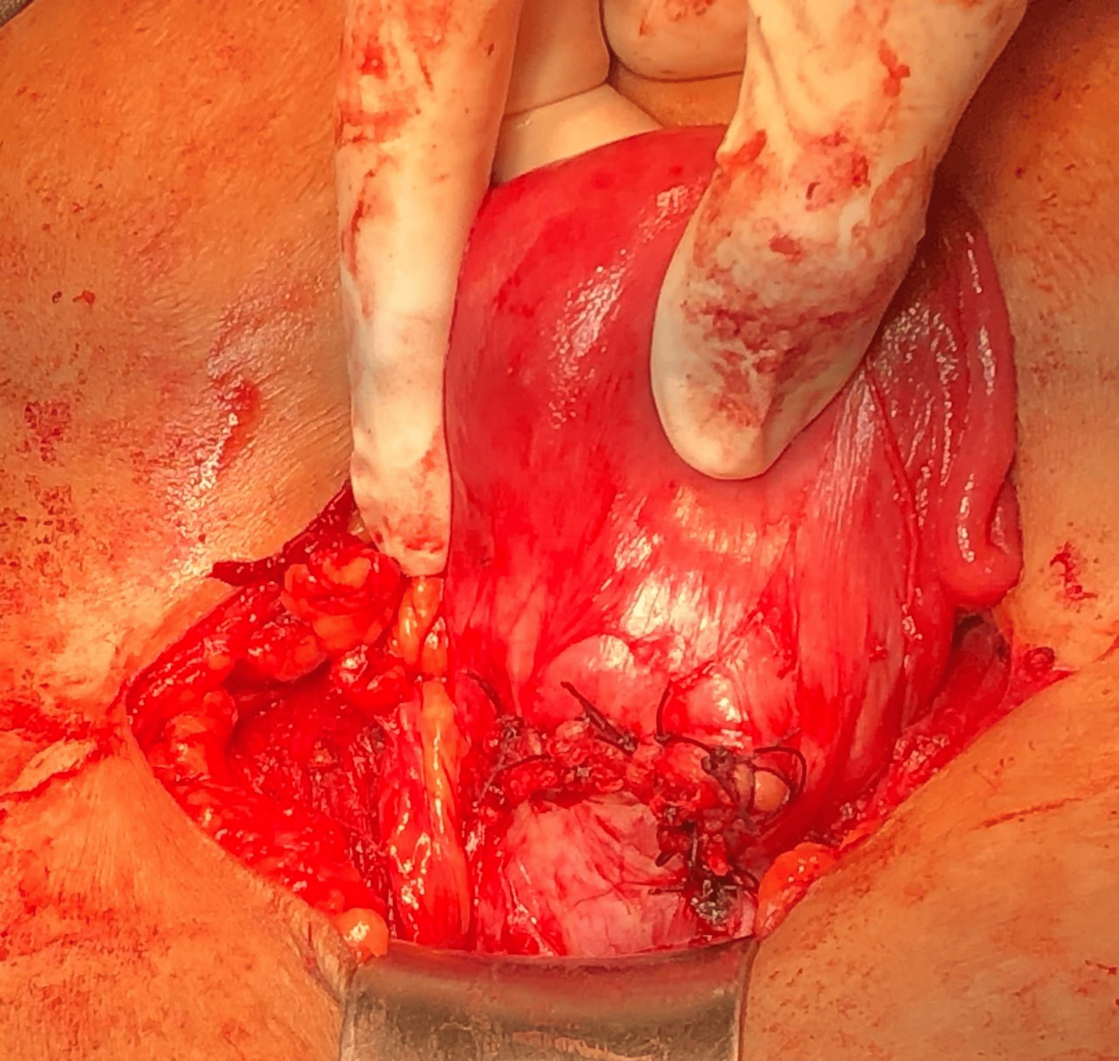
Scar area post-repair.

The uterus was bulky, with the lower uterine segment bulging, vascular, and distended. Foul-smelling, necrotic products of conception were evacuated from the lower uterine segment. Both fallopian tubes and ovaries appeared normal. A small incision was made to the uterus at the site of the bluish, bulging membrane, and all the products of conception were removed (Figures 2A-2C). The previous scar tissue was excised. The uterine incision was then closed using Vicryl interlocking sutures (Ethicon, Inc., Bridgewater, USA) (Figure 3). The intraoperative period was uneventful. The histopathology report was consistent with products of conception with deciduitis.

Postoperative course

**Figure 4:**
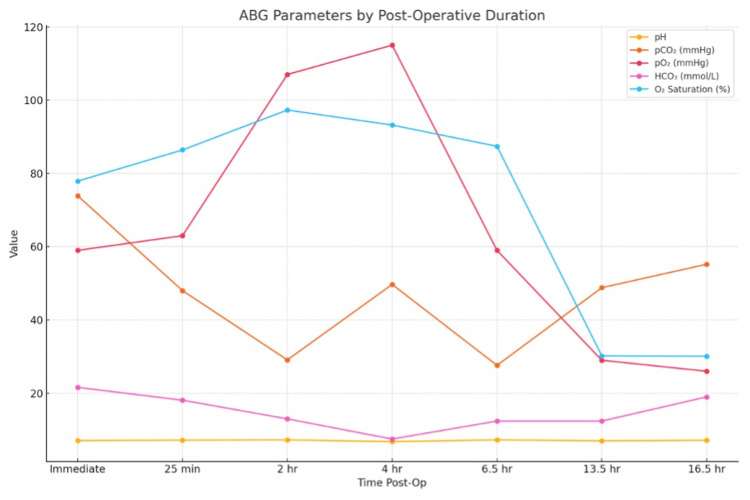
Blood gas parameters and trends. ABG: arterial blood gas; pCO_2_: partial pressure of carbon dioxide; pO_2_: partial pressure of oxygen; HCO_3_: bicarbonate; O_2_ saturation: oxygen saturation

Postoperatively, the patient was extubated; however, she developed hypoxia, with oxygen saturation dropping to 80% despite receiving oxygen via face mask at 6 L/min in the recovery room. Due to worsening respiratory distress at around one hour postoperatively, the patient was taken to the ICU and was re-intubated due to her deteriorating status immediately. She was commenced on inotropic support and continued to deteriorate clinically. Serial arterial blood gas (ABG) analysis demonstrated severe metabolic acidosis (Table 2 and Figure 4). Approximately three hours after surgery, the patient developed bradycardia and hypotension despite inotropic support. Cardiopulmonary resuscitation (CPR) was initiated, and return of spontaneous circulation (ROSC) was achieved. A rapid decline in oxygen saturation and PaO_2_ levels strongly suggested acute respiratory failure, likely secondary to sepsis-induced multiorgan dysfunction syndrome (MODS). Liver function tests revealed significant derangement, consistent with ischemic hepatitis, showing elevated enzymes: aspartate transaminase levels (AST): 10458 units/L and alanine transaminase (ALT): 5982 units/L. Blood gas analysis showed severe metabolic acidosis with persistent hypoxia. White cell count was elevated (up to 67,900/mm^3^), creatinine was raised (2.1 mg/dL), severe thrombocytosis, and a deranged prothrombin time were noted.

**Table 2:** Timeline of arterial blood gas (ABG) values of the patient pCO_2_: partial pressure of carbon dioxide; pO_2_: partial pressure of oxygen; HCO_3_: bicarbonate; O_2_ saturation: oxygen saturation

ABG	Time post-operative	pH	pCO_2_ (mmHg)	pO_2_ (mmHg)	HCO_3_ (mmol/L)	O_2_ saturation (%)
ABG 1	Immediate	7.08	73.9	59	21.6	77.9
ABG 2	25 min	7.18	48.0	63	18.1	86.4
ABG 3	2 hr	7.26	29.1	107	13.0	97.3
ABG 4	4 hr	6.79	49.7	115	7.5	93.2
ABG 5	6.5 hr	7.26	27.6	59	12.4	87.4
ABG 6	13.5 hr	7.01	48.8	29	12.4	30.2
ABG 7	16.5 hr	7.15	55.2	26	19.0	30.1

The overall diagnosis included septic shock with MODS, rheumatic valvular heart disease (RVHD) with severe MS, PAH, and fulminant hepatitis. Given the refractory PAH, intravenous milrinone was administered. An arterial line was secured for invasive hemodynamic monitoring; however, the patient’s condition remained critical and progressively worsened. After detailed discussions regarding the poor prognosis, the family provided do-not-resuscitate (DNR) consent. Despite aggressive medical management, the patient ultimately succumbed to her illness.

## Discussion

Pregnancy places additional hemodynamic stress on the cardiovascular system, which may unmask previously undiagnosed conditions such as MS. In RHD, MS is the most common manifestation. The inability of the narrowed mitral valve to accommodate the increased blood volume of pregnancy leads to elevated left atrial pressures, often resulting in pulmonary oedema and breathlessness. Echocardiography remains essential for assessing the severity of MS and complications such as pulmonary hypertension [4,5].

CSP is defined as implantation of a pregnancy at the site of a previous lower uterine segment caesarean section scar. The underlying pathophysiology remains debated, but proposed mechanisms include embryo migration through a scar defect or trophoblastic invasion at a weakened myometrial site. Two types of CSP have been classified: (1) on-the-scar (type 1), where implantation occurs on a healed scar; and (2) in-the-niche (type 2), where implantation occurs within the defect of a poorly healed scar [6-8]. Specifically, type 1, or on-the-scar, refers to the implantation of the gestational sac on the well-healed scar of a previous caesarean section (also termed “endogenous” implantation) [9,10]. Type 1 CSPs usually have a myometrial thickness of ≥3 mm between the placenta and the bladder or anterior uterine serosa. Type 2 has implantation of the gestational sac within the defect or “niche” of an incompletely healed scar (also termed “niche pregnancy” or “exogenous” implantation). Type 2 CSPs typically show a thin (<3 mm) or absent myometrial layer between the placenta and the bladder or anterior uterine serosa.

Sepsis, septic shock, and MODS represent a continuum of systemic inflammatory and organ dysfunction responses to infection. According to the Sepsis-3 definitions established by the Third International Consensus, sepsis is defined as life-threatening organ dysfunction caused by a dysregulated host response to infection, operationalised as an increase of ≥2 points in the Sequential Organ Failure Assessment (SOFA) score from baseline, in the presence of suspected or confirmed infection. Septic shock is considered a severe subset of sepsis, characterised by persistent hypotension requiring vasopressor therapy to maintain a mean arterial pressure (MAP) of ≥65 mm Hg and a serum lactate level >2 mmol/L despite adequate fluid resuscitation. MODS, on the other hand, is conceptualised as the progressive dysfunction of two or more organ systems following a systemic insult such as sepsis, often quantified using SOFA sub-scores or similar composite scoring systems. While no single universally accepted definition for MODS exists, its recognition is critical in prognostication and guiding intensive care interventions. Together, these criteria underscore the importance of early identification and organ-supportive therapy in the management of critically ill patients with sepsis-spectrum disorders [11,12].

This case represents a complex clinical scenario involving a young woman with multiple high-risk conditions: RVHD with severe MS, PAH, HFpEF, and a scar ectopic pregnancy. She had self-administered MTP pills without medical supervision. Misoprostol, a prostaglandin E1 analogue, is known to cause side effects such as tachycardia, hypotension, fever, and abdominal cramping. Unsupervised MTP poses significant risks, including severe haemorrhage, sepsis, and hypovolemic shock.

In the context of RHD, such complications can precipitate acute cardiac decompensation and profound hemodynamic instability. Furthermore, due to medicolegal concerns, patients often delay seeking timely medical attention, which can result in poor outcomes, particularly in individuals with compromised cardiac function. Before initiating MTP, it is imperative to confirm intrauterine gestation and exclude ectopic pregnancy. In this case, the patient, with a history of one prior LSCS, had consumed abortifacients without prior imaging or clinical evaluation, thereby significantly increasing her risk of mortality.

The clinical course was further complicated by infection, as evidenced by foul-smelling vaginal discharge and a culture-positive vaginal swab, ultimately progressing to septic shock. Her cardiac comorbidities - severe MS and PAH - significantly increased the perioperative risk. The stress of surgery, combined with the burden of sepsis, led to the development of MODS. Fulminant hepatitis, evidenced by massively elevated liver enzymes (AST >10,000 U/L), was likely the result of a combined septic and ischemic insult. Despite exploratory laparotomy and optimal treatment, intensive care unit (ICU) management, administration of broad-spectrum antibiotics, and multispecialty involvement, the patient’s condition rapidly deteriorated. The prognosis in cases of overlapping critical illnesses - including infectious, cardiovascular, hepatic, renal, and obstetric pathologies - is exceedingly poor. Multiple episodes of cardiac arrest and bradycardia marked the patient’s steady clinical decline.

According to the Royal College of Obstetricians and Gynaecologists (RCOG) criteria, the diagnosis of CSP is based on the following features: First, the uterine cavity is empty. Second, the gestational sac or a solid trophoblastic mass is visualised anteriorly at the level of the internal os, specifically embedded at the site of the previous lower uterine segment caesarean scar. Third, the myometrial layer between the gestational sac and the bladder is notably thin or absent, increasing the risk of bladder invasion or uterine rupture. Fourth, Doppler examination shows prominent trophoblastic or placental circulation, reflecting active and abnormal vascularisation at the scar site. Finally, the endocervical canal remains empty, ruling out a cervical pregnancy. Doppler imaging is often instrumental, revealing increased vascularity surrounding the trophoblastic tissue [13,14].

Management of CSP must be individualised based on the severity of symptoms, reproductive desires, gestational age, serum hCG levels, and myometrial thickness [13]. Medical therapy, including systemic or local administration of methotrexate, is suitable for hemodynamically stable patients. According to RCOG guidance, methotrexate is particularly effective when the gestational age is under eight weeks and hCG levels are below 5000 IU/L [14]. Intra-lesional administration of methotrexate offers a higher success rate and can be considered in stable patients who meet selection criteria [15,16].

Surgical management is reserved for patients presenting with significant clinical symptoms or those who decline medical treatment. For endogenous (type 1) CSPs, hysteroscopic or suction evacuation is generally safer and carries less risk of uterine perforation. For exogenous (type 2) CSPs, where the myometrium is thin or absent, surgical excision followed by uterine repair (resuturing) is often indicated [17,18]. Overall, expectant management is rarely advised due to the high risk of severe complications, including haemorrhage, uterine rupture, and maternal morbidity.

## Conclusions

This case underscores the dangers associated with unsupervised MTP. CSP, particularly in women with a history of LSCS, can lead to life-threatening complications. Early recognition of high-risk comorbidities, such as RHD and PAH, during pregnancy is crucial and necessitates a multidisciplinary approach for optimal management. Despite optimal treatment, it might be difficult to save a patient in severe sepsis and multiorgan dysfunction. Also highlighting the timely referral of these patients to a tertiary centre. The self-administration of MTP pills should be strongly discouraged, especially in the absence of prior imaging and clinical evaluation.
